# Spontaneous remission in adult patients with IgA nephropathy treated with conservative therapy

**DOI:** 10.1371/journal.pone.0251294

**Published:** 2021-05-27

**Authors:** Hirotaka Sato, Daisuke Ichikawa, Eri Okada, Tomo Suzuki, Shiika Watanabe, Sayuri Shirai, Yugo Shibagaki

**Affiliations:** 1 Division of Nephrology and Hypertension, Department of Internal Medicine, St. Marianna University School of Medicine, Kanagawa, Japan; 2 Kidney Center, National Hospital Organization Chiba-East Hospital, Chiba, Japan; 3 Department of Nephrology, Kameda Medical Center, Chiba, Japan; 4 Division of Nephrology and Hypertension, Department of Internal Medicine, St. Marianna University School of Medicine, Yokohama City Seibu Hospital, Kanagawa, Japan; University of Glasgow, UNITED KINGDOM

## Abstract

**Background:**

There are few studies describing the clinical course and spontaneous remission of IgA nephropathy (IgAN) in adult patients receiving conservative treatment.

**Method:**

Data from 62 adult patients with biopsy-diagnosed IgAN, who received conservative treatment at least 5 years prior, were retrospectively investigated. No patients received corticosteroids, other immunosuppressants, or tonsillectomy. Remission of proteinuria and hematuria were defined as proteinuria <0.3 g/gCr and urine red blood cells (RBC) <5 / high power field (HPF) on three consecutive urinalyses obtained during an observation period of ≥6 months.

**Result:**

Thirty-eight (61.3%) patients had remission of hematuria, 24 (38.7%) had remission of proteinuria, and 19 (30.6%) had remission of both. Remission rates increased in patients with proteinuria <0.5 g/g Cr at diagnosis. The median time to remission of hematuria was 2.8 years and that of proteinuria was 2.6 years. Patients who showed renal function decline (defined as 30% decline of estimated glomerular filtration rate [eGFR] from baseline) were older, had significantly lower eGFR, and higher proteinuria at diagnosis. Two patients with preserved renal function and normal proteinuria at diagnosis experienced renal function decline. Renal function did not decline within 3 years of diagnosis in patients with proteinuria <1 g/gCr at diagnosis.

**Conclusions:**

Relatively high rates of spontaneous remission were observed. Remission of both hematuria and proteinuria were frequent within 3 years after diagnosis, and renal function was well preserved during this period. These data indicate that it is rational to use conservative treatment for 3 years after the diagnosis instead of aggressive treatments.

## Introduction

IgA nephropathy (IgAN) is the most common primary glomerulonephritis [1]. The renal survival rate of adults with IgAN is approximately 80% [2], through some cases progress to ESKD, meaning both appropriate treatment and careful follow-up are required.

Clinical presentations of IgAN are highly variable, and the treatment strategy is mainly based on histologic and clinical severity at diagnosis, which includes observation, administration of renin-angiotensin system (RAS) inhibitors, antiplatelet agents, prednisolone (PSL) and other immunosuppressive drugs, and bilateral tonsillectomy [3, 4].

The treatment strategy of IgAN with a clinically benign presentation at diagnosis is different between institutions, and is not clearly established. While there are some reports of spontaneous remission and favorable prognosis of clinically benign IgAN in pediatric studies [5–7], there are few reports in adults.

We often observe spontaneous remission patients with conservative treatment alone, but the specific clinical course and renal prognosis are not well documented. It is controversial whether aggressive treatment should be used for such patients

If adult IgAN with benign presentation at diagnosis is not exacerbated after conservative treatment alone, aggressive treatment such as administration of corticosteroids, immunosuppressants, and tonsillectomy may not be necessary, but the details of clinical remission in such patients is unclear.

Randomized clinical trials of IgA nephropathy treated with immunosuppressive therapy have reported serious side effects of infection, so if the disease does not progress, immunosuppressive therapy should be avoided [8].

Therefore, we retrospectively investigated the clinical course of adult patients with IgAN who received conservative treatment alone to elucidate the details of clinical remission and long-term renal outcomes.

## Materials and methods

### Patients and clinical data

This study was performed in accordance with the principles of the Declaration of Helsinki, and ethical approval was obtained from the ethics committee of St. Marianna university school of medicine. Clinical information was collected from computerized medical records and fully anonymized. The ethics committee waived the requirement for informed consent. Adult patients, aged > 20 years, who were newly diagnosed with IgAN by renal biopsy between 1 January 2000 and 31 December 2013 were included in this study. Patients with secondary IgAN were excluded. Patients visited our department at intervals of 3–6 months for at least 5 years. Patients who received either corticosteroids, other immunosuppressants, or tonsillectomy during the follow-up period were excluded.

A total of 369 patients were diagnosed with primary IgAN by renal biopsy in this period, of which 127 patients were followed up for at least 5 years. 65 of 127 patients with aggressive treatments were excluded (tonsillectomy and methylprednisolone pulse in 41 patients, methylprednisolone pulse in 15 patients, oral prednisolone in 8 patients, oral prednisolone and cyclophosphamide in one patient). Ultimately, 62 patients met the inclusion criteria. Each patient was treated with conservative therapy alone (RAS inhibitors, antiplatelet drugs, or no medication).

We collected demographic and clinical data including sex, age, follow-up duration, the use of RAS inhibitors (ARBs or ACE-inhibitors) or antiplatelet drugs, serum creatinine (sCr), estimated glomerular filtration rate (eGFR), urinary protein (UP, defined by urinary protein-to-creatinine ratio), urine sediment, serum IgA, and pathological characteristics.

### Outcomes

The primary outcome was clinical remission. Absence of proteinuria was defined as a urinary protein/creatinine ratio < 0.3 g/gCr, and that of hematuria is defined as urine red blood cell < 5 /HPF in urine sediment respectively.

Remission was defined if urinalysis results met the above each normalization criteria three times consecutively during an observation period of at least 6 months, in reference to past research [9]. The date of remission was defined as the first day of the above three times.

The secondary outcome was renal function decline, defined as a 30% decrease of eGFR from the time of renal biopsy, according to the KDIGO proposal of endpoint of renal disease [10]. ESKD was defined as an eGFR < 15 mL/min/1.73m^2^. eGFR was estimated by the Modification of Diet in Renal Disease equation [11].

### Pathology

We reviewed each biopsy specimen and re-evaluated 4 pathological features including mesangial hypercellularity (M1), segmental glomerulosclerosis including adhesion (S1), endocapillary hypercellularity (E1), and interstitial fibrosis (T1-2) according to the Oxford classification [12, 13]. In addition, we also re-evaluated for crescent formation and global glomerulosclerosis.

### Statistical analyses

Normally distributed data are expressed as the mean ± standard deviation (SD). Non-normally distributed data are expressed as the median and interquartile range (IQR). Continuous variables were analyzed using the Student’s t-test in the case of normal distribution, and Mann-Whitney U test in the case of non-normal distribution. The Fisher’s exact test was used to compare ratios between the two groups.

Cumulative event rates, such as renal function decline and clinical remission, were calculated using the Kaplan–Meier method. Univariate regressions were used to identify the variables related to the secondary outcome. Variables showing P values < 0.05 in univariate tests, variables related to primary outcome identified in past studies (RAS inhibitor use, age, proteinuria at diagnosis) and baseline eGFR, were used for the multivariate model. All P-values were two-sided, and P-values of <0.05 were considered statistically significant. All statistical analyses were performed with EZR (Saitama Medical Center, Jichi Medical University, Saitama, Japan), which is a graphical user interface for R (The R Foundation for Statistical Computing, Vienna, Austria, version 1.6–3) [14]. More precisely, it is a modified version of R commander (version 1.6–3) designed to add statistical functions frequently used in biostatistics.

## Results

### Patient characteristics

There were 62 patients, 36 men and 26 women (Table 1). The mean age was 39.9 ± 12.4 years. The median observation period was 9.9 years (IQR 6.1–12.9). RAS inhibitors were used in 51 patients (82.3%). ARBs were used in 47 patients (75.8%), ACE-inhibitors in 15 patients (24.2%) and combination of both in 11 patients (17.7%). Antiplatelet drugs (Dipyridamole or Dilazep hydrochloride hydrate) were used in 11 patients (19.3%). All patients using antiplatelet drugs also used RAS inhibitors.

**Table 1:** Clinical characteristics of included patients (n = 62).

Variables	Values
Age (years)	39.9 ± 12.4
Men	36 (58%)
Follw-up duration (years)	9.9 (6.1–12.9)
RAS inhibitors	51 (82.3%)
Antiplatelet drugs	11 (19.3%)
sCr (mg/dL)	0.89 ± 0.26
Baseline eGFR (mL/min/1.73 m^2^)	75.6 ± 23.2
Baseline proteinuria (g/gCr)	0.23 (0.18–0.57)
< 0.5	43 (69.4%)
0.5–1	10 (16.1%)
> 1	9 (14.5%)
Hematuria	62 (100%)
Baseline IgA (mg/dL)	352.0 ± 102.7
MEST score	
M1	39 (62.9%)
E1	1 (1.6%)
S1	29 (46.8%)
T1-2	8 (12.9%)
Crescent formation	16 (25.8%)
fibrous	3 (4.8%)
cellular or fibrocellular	13 (21.0%)
Global sclerosis	46 (74.2%)

The mean eGFR at renal biopsy was 75.6 ± 23.2 mL/min/1.73m^2^. There were 19 patients with an eGFR < 60 mL/min/1.73m^2^ at renal biopsy. All patients had hematuria. The median amount of proteinuria at diagnosis was 0.23 g/gCr (IQR 0.18–0.57). Proteinuria of > 1 g/gCr (n = 9), 0.5–1 g/gCr (n = 10), and < 0.5 g/gCr (n = 43) were observed.

### Primary outcome

38(61.3%) patients had remission of hematuria, 24(38.7%) patients had remission of proteinuria, and 19 (30.6%) patients had remission of both (Table 2).

**Table 2:** Clinical remission by amount of proteinuria.

	All (n = 62)	UP < 0.5 g/gCr (n = 43)	UP 0.5–1.0 g/gCr (n = 10)	UP > 1g/gCr (n = 9)
Hematuria remission	38 (61.3%)	29 (67.4%)	6 (60%)	3 (33.3%)
Proteinuria remission	24 (38.7%)	21 (48.8%)	1 (10%)	2 (22.2%)
Both remission	19 (30.6%)	18 (41.9%)	0 (0%)	1 (11.1%)

**Fig 1:**
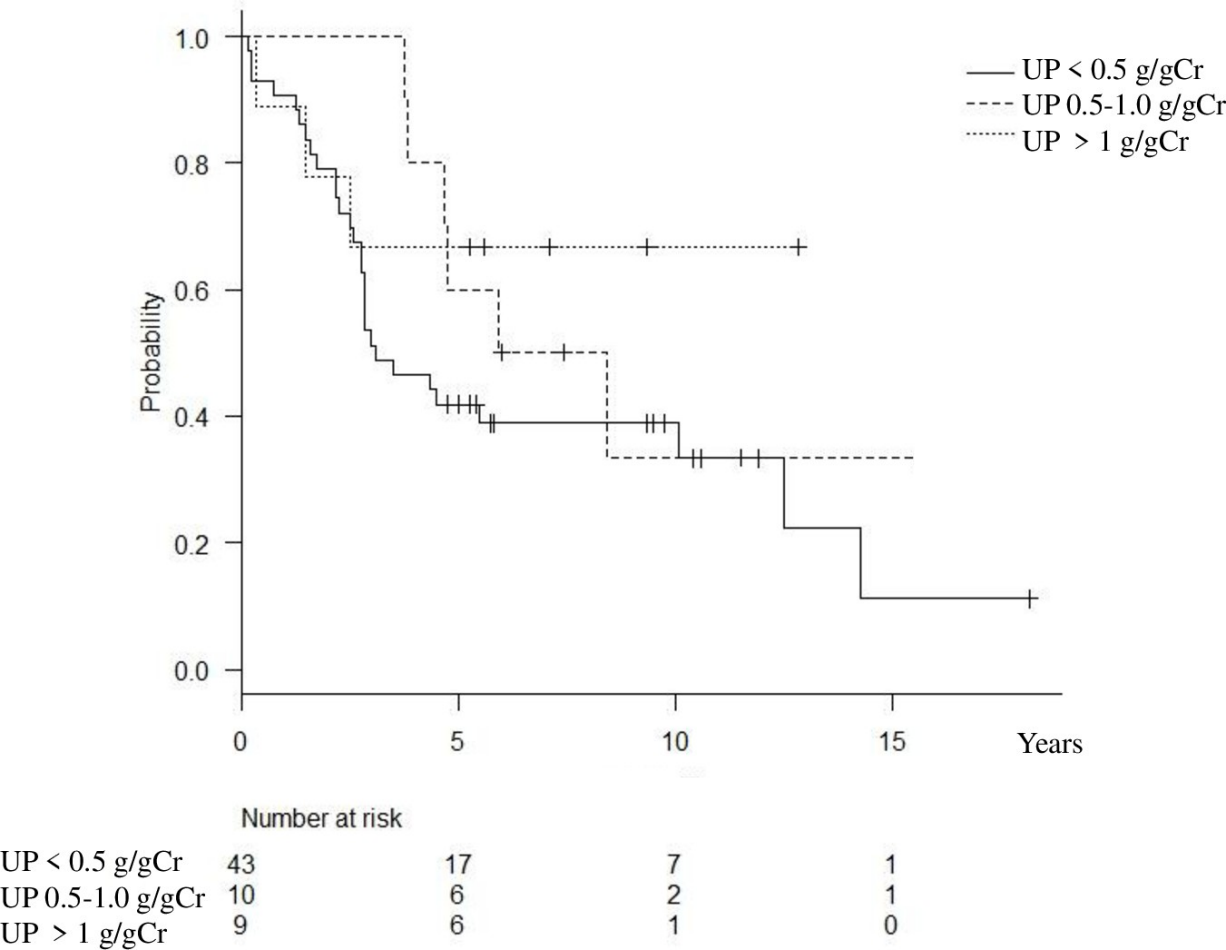
Kaplan-Meier plot of hematuria remission, defined by disappearance of microscopically visible urine red blood cells <5 /HPF in urine sediment.

**Fig 2:**
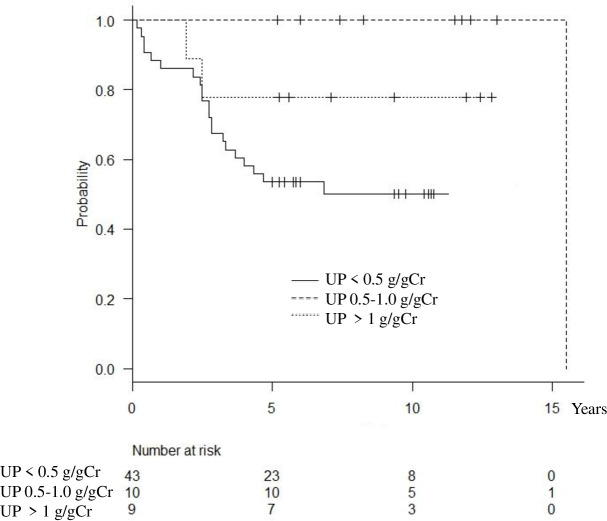
Kaplan-Meier plot of proteinuria remission, defined by persistent proteinuria of <0.3 g/gCr.

Remission rate for hematuria and proteinuria increased in the patients with proteinuria of < 0.5 g /g Cr at diagnosis. The median time to hematuria remission was 2.8 years (IQR 1.6–4.2), and that of proteinuria was 2.6 years (IQR 1.7–3.4) for the group with proteinuria of < 0.5 g/gCr at diagnosis (Figs 1 and 2).

In the univariate analysis, proteinuria of < 0.5 g/gCr at diagnosis was identified as an independent potential factor for hematuria remission within 3 years. In the multivariate analysis, proteinuria of < 0.5 g/gCr at diagnosis (odds ratio [OR] 0.20, 95% CI 0.05–0.82, P = 0.03) remained an independent potential factor (Table 3). We did not identify any factors associated with proteinuria remission within 3 years by univariate or multivariate analysis.

**Table 3:** Univariate and multivariate analysis of independent potential factors for remission of hematuria within 3 years.

	Univariate Analysis		Multivariate Analysis	
	Hazard Ratio (95% CI)	P value	Hazard Ratio (95% CI)	P value
Age < 40 versus > 40	1.88 (0.66–5.31)	0.24	2.3 (0.55–9.64)	0.26
Baseline eGFR > 60 mL/min/1.73 m^2^ versus < 60 mL/min/1.73 m^2^	1.23 (0.41–3.75)	0.71	0.55 (0.12–2.5)	0.44
Baseline proteinuria < 0.5 g/gCr versus > 0.5 g/gCr	5.60 (1.42–22.0)	0.01	5.79 (1.38–24.3)	0.02
M0 versus M1	2.92 (1.0–8.52)	0.05	2.35 (0.63–8.71)	0.2
RAS inhibitors use Yes versus No	0.36 (0.08–1.66)	0.19	0.66 (0.1–4.26)	0.66

### Secondary outcome

**Fig 3:**
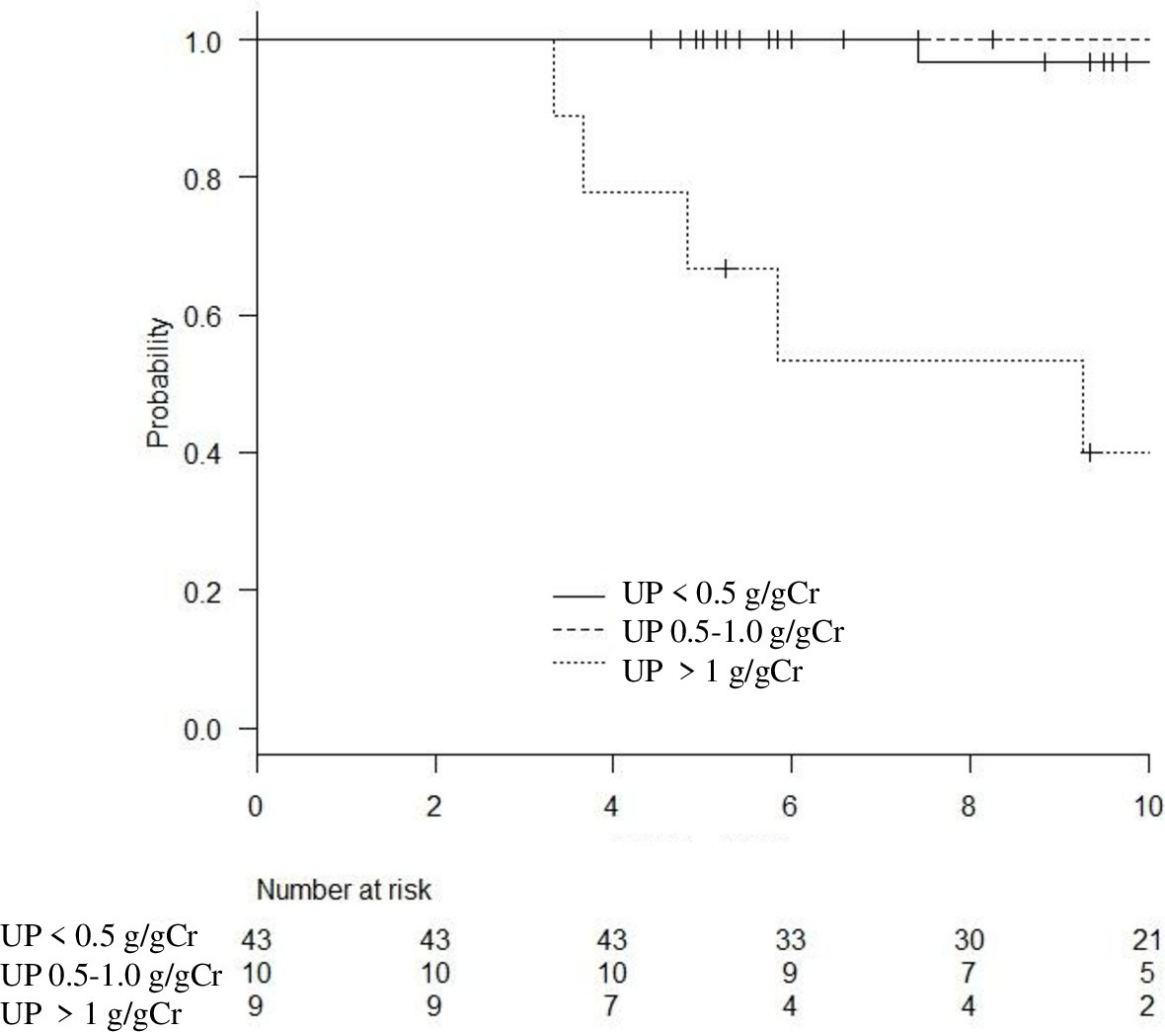
Renal survival probability, defined by less than 30% eGFR decline from diagnosis, as determined by Kaplan–Meier analysis.

Renal survival rates (eGFR decline <30%) after 5 and 10 years from diagnosis were 95.1% and 88.6%, respectively. Renal function declined in 10 patients (progressed group), of which 3 cases progressed to ESKD. In patients with proteinuria of < 1.0 g/gCr at diagnosis, renal function was well preserved for 3 years after the diagnosis (Fig 3).

Patients in the progressed group were older, with lower eGFR, and higher proteinuria at diagnosis compared to patients with preserved renal function (preserved group). Pathologically, the S and T lesions were more common in the progressed group (Table 4).

**Table 4:** Comparison of baseline characteristics between preserved group and progressed group.

Variable	Preserved group (n = 52)	Progressed group (n = 10)	P value
Age (years)	38.4 ± 11.8	47.7 ± 13.8	0.03
Men	30 (58%)	6 (60%)	1
Follw-up duration (years)	9.6 (6–12.3)	12.1 (8–13.5)	0.28
RAS inhibitor	41 (78.8%)	10 (100%)	0.329
antiplatelet drug	10 (19.2%)	2 (20%)	1
Baseline eGFR (mL/min/1.73 m^2^)	78.8 ± 23.1	58.5 ± 17.6	0.01
Baseline proteinuria (g/gCr)	0.21(0.17–0.41)	1.5(0.46–2.78)	0.003
Baseline IgA (mg/dL)	354.6 ± 106.9	336.9 ± 85.4	0.64
MEST score			
M1	31 (59.6%)	8 (80%)	0.3
E1	0 (0%)	1 (10%)	0.161
S1	21 (40.4%)	8 (80%)	0.04
T1-2	4 (7.7%)	4 (40%)	0.02
Crescent formation	12 (23.1%)	4 (40%)	0.26
fibrous	1 (1.9%)	2 (20%)	0.07
cellular or fibrocellular	11 (21.2%)	2 (20%)	1
Global sclerosis	37 (71.2%)	9 (90%)	0.43

Two patients in the progressed group had normal renal function and normal proteinuria at diagnosis (Table 5). Both patients received RAS inhibitors, and M and S lesions were observed on renal biopsy. In patient 1, proteinuria did not increase, and hematuria remission was continuously obtained during the follow-up period. However, the patient experienced a gradual deterioration of renal function, and 30% GFR decline was observed 12.1 years after diagnosis. In patient 2, hematuria remission was obtained 10 years after renal biopsy, but proteinuria gradually increased during follow-up, and 30% GFR decline was seen 7.4 years after renal biopsy. Neither patient has progressed to ESKD.

**Table 5:** Clinical characteristics of two patients with benign presentation at diagnosis who showed renal function decline.

Variable	Patient 1	Patient 2
Age (years)	22	41
Sex	male	female
Follw-up duration (years)	18.3	10.8
duration to renal function decline	12.1	7.4
RAS inhibitor	Yes	Yes
antiplatelet drug	No	No
Baseline eGFR (mL/min/1.73 m^2^)	79.9	67.6
Baseline proteinuria (g/gCr)	0.18	0.26
Baseline IgA (mg/dL)	403	356
MEST score	M1E0S1T0	M1E0S1T1
Crescent formation	Yes	No
Global sclerosis	No	Yes

## Discussion

As a result of long-term investigation in patients with IgA nephropathy who underwent conservative therapy, we showed that spontaneous remission occurred at relatively high rate within a few years.

The prognosis of patients with mild IgAN who received conservative treatment alone has been well described in pediatric studies. A high rate of spontaneous remission in patients with IgAN and minor glomerular abnormalities or focal mesangial proliferation was reported [5]. These patients received no medication, and 57 patients (59.4%) achieved remission. The mean time to remission was 5.9 ± 0.4 years. Another study analyzed 106 IgAN patients with mild proteinuria (<0.5 g/day), of which 4 patients received immunosuppressive therapy, and reported that no patients progressed CKD within 15 years after onset [6]. A high rate of proteinuria resolution at 24 months after RAS inhibitor treatment was also reported [7].

Similar to our study, a study in Spain reported the long-term clinical course of 141 Caucasian IgAN patients with preserved renal function and minimal (< 0.5 g/day) or no proteinuria at diagnosis [15]. These patients received conservative therapy or no medication, and clinical remission was achieved in 53 patients (37.5%). The median time to remission was 48 months. Only 5 patients showed a 50% increase of sCr from baseline, and no patients progressed to ESKD. In a Japanese study of 20 IgAN patients with normal renal function and proteinuria of < 0.5 g/day at diagnosis, the 15-year renal survival rate was 93.8%, and clinical remission was observed in 9 (45%) patients. Baseline proteinuria was associated with the absence of clinical remission [16]. Spontaneous remission, even in adult patients with IgAN with nephrotic syndrome, was reported in 2011 [17]. In this report, authors suggested careful observation without immunosuppressive therapy. These studies suggest good long-term prognosis of mild IgAN.

In contrast to these studies, Lee et al. analyzed 153 IgAN patients with preserved renal function and proteinuria < 0.5 g/day who received no immunosuppressive drugs in 2014. Six patients developed ESKD, and the 30-year renal survival rate was 85.5%. They concluded that the long-term prognosis of clinically mild IgAN is not always favorable [18]. A study of 74 IgAN patients treated with conservative therapy between 1968 and 1974 [19], found that 28 patients (37.8%) showed elevation of sCr > 1.36 m/dL during the follow-up period, and 22 patients (37.8%) progressed to ESKD. Shen et al. retrospectively studied 135 IgAN patients with isolated microscopic hematuria, with a mean follow-up period of 92 ± 28 months. They reported remission of hematuria in 16 patients (12%), emergence of proteinuria in 39 patients (29%), and 227 patients (20%) developed renal insufficiency (GFR < 60/mL/min/1.73 m^2^) [20].

In our study, remission of hematuria and proteinuria in adult IgAN patients who were relatively young and had clinically benign presentation at renal biopsy was frequently seen within 3 years after renal biopsy, and the renal function was also well preserved in this follow-up period. In contrast, patients with higher proteinuria (> 1g/gCr) at diagnosis have a high probability of progression to renal failure as reported in past study [21]. Only one patient in this group obtained both proteinuria and hematuria remission. In addition, patients who experienced renal function decline were relatively older at diagnosis. Aging was previously reported to promote IgAN progression [22].

From these results, we suggest that 3 years of observation with conservative, instead of aggressive, treatment is sufficient to expect spontaneous remission in young patients with benign IgAN.

However, two of the ten patients in the progressed group had a benign presentation at diagnosis. In one patient, remission of both hematuria and proteinuria was obtained, but the eGFR declined gradually, and reached a 30% decline from baseline 12.1 years after renal biopsy. This case showed crescent formation and segmental glomerulosclerosis, which indicated poor renal prognosis pathologically. Several factors are reported as risk of renal failure progression in IgAN patients with benign presentation at diagnosis. A previous study investigated benign IgAN that was detected early in patients treated conservatively, and those who progressed to ESKD had advanced-grade pathological features, including segmental sclerosis, tubular atrophy, and interstitial fibrosis [18]. Tubulointerstitial lesion is also reported to be useful marker of poor renal outcome in IgAN patients presented with normal blood pressure, preserved renal function and proteinuria < 0.4 g/gCr at diagnosis [23]. In addition, baseline age, proteinuria, and Oxford T lesion were reported to be risk factors of worse renal outcome in primary IgA patient with proteinuria <1.0 g/day at diagnosis [24].

Importantly, remission of hematuria was reported to be associated with better renal prognosis [23, 25], and proteinuria increase was associated with poor renal prognosis [26, 27].

From these studies, we recommend careful observation especially for patients with these pathological and clinical risk factors. Effectiveness of early initiation of glucocorticoids even in IgAN patients with minimal proteinuria at diagnosis is reported [28], so prompt initiation of aggressive treatments is also important as soon as the clinical progression is observed during follow-up even if the patient has the mild presentation at diagnosis.

Our study has several limitations. The mode of onset and the duration from onset to renal biopsy were unclear. The duration from onset of IgAN to renal biopsy is reported to be associated with clinical and pathological severity [29]. We could not examine the presence or absence of hypertension, dyslipidemia, hyperuricemia, or other comorbidities at renal biopsy. Proteinuria was not assessed by 24-hour urine collection. Some patients ceased to follow up after 5 years from renal biopsy, and we could not follow all cases in the long term.

In conclusion, the clinical course of adult IgAN patients who are young, with preserved renal function and mild proteinuria at the time of renal biopsy, is relatively good, and clinical remission was frequently seen within 3 years after diagnosis. Hence, it is rational to expect spontaneous remission within 3 years after diagnosis with conservative treatment instead of aggressive treatment in such patients.

## Supporting information

S1 Data (XLSX)
